# Detecting the impact of diagnostic procedures in Pap-positive women on anxiety using artificial neural networks

**DOI:** 10.1371/journal.pone.0312870

**Published:** 2024-10-31

**Authors:** Irena Ilic, Goran Babic, Aleksandra Dimitrijevic, Sandra Sipetic Grujicic, Vladimir Jakovljevic, Ivana Zivanovic Macuzic, Milena Ilic

**Affiliations:** 1 Faculty of Medicine, University of Belgrade, Belgrade, Serbia; 2 Faculty of Medical Sciences, Department of Gynecology and Obstetrics, University of Kragujevac, Kragujevac, Serbia; 3 Faculty of Medicine, Institute of Epidemiology, University of Belgrade, Belgrade, Serbia; 4 Faculty of Medical Sciences, Department of Physiology, University of Kragujevac, Kragujevac, Serbia; 5 Faculty of Medical Sciences, Department of Anatomy, University of Kragujevac, Kragujevac, Serbia; 6 Faculty of Medical Sciences, Department of Epidemiology, University of Kragujevac, Kragujevac, Serbia; Teikyo University, School of Medicine, JAPAN

## Abstract

**Introduction:**

Women who receive a result of an abnormal Papanicolaou (Pap) smear can fail to participate in follow up procedures, and this is often due to anxiety. This study aimed to apply artificial neural networks (ANN) in prediction of anxiety in women with an abnormal Pap smear test, prior to and following diagnostic procedures.

**Methods:**

One hundred-seventy two women who received an abnormal Pap screening result took part in this study, completing a questionnaire about socio-demographic characteristics and Hospital Anxiety and Depression Scale (HADS), right before and two to four weeks after diagnostics (i.e. colposcopy/biopsy/endocervical curettage). A feedforward back-propagation multilayer perceptron model was applied in analysis.

**Results:**

Prior to diagnostic procedures 50.0% of women experienced anxiety, while after diagnostics anxiety was present in 61.6% of women. The correlation-based feature selection showed that anxiety prior to diagnostic procedures was associated with the use of sedatives, worry score, depression score, and score for concern about health consequences. For anxiety following diagnostics, predictors included rural place of residence, depression score, history of spontaneous abortion, and score for tension and discomfort during colposcopy. The ANN models yielded highly accurate anxiety prediction both prior and after diagnostics, 76.47% and 85.30%, respectively.

**Conclusion:**

The presented findings can aid in identification of those women with a positive Pap screening test who could develop anxiety and thus represent the target group for psychological support, which would consequently improve adherence to follow-up diagnostics and enable timely treatment, finally reducing complications and fatal outcome.

## Introduction

More than 600,000 incident cases (6.5% of all) and almost 340,000 deaths (7.7% of all) of cancer in women in 2020 worlwide, rank cervical cancer as the number four most common cancer in women [1,2]. In 2020, Asia recorded most (nearly 60%) of the world’s cases of cervical cancer, followed by Africa with 20%, while North America recorded about 2%. Countries suffering from having limited resources, such as Serbia, still face cervical cancer as a serious public health problem [2,3]. Every year in Serbia, around 1300 women get cervical cancer and about 500 women die from this disease [1,3]. In Serbia, cervical cancer ranks as the number three most common cancer in women [2,3]. In 2020, cervical cancer incidence rate (per 100,000 population) in Serbia was 18.7, higher than the rates reported in developed countries: 6.2 in the United States, 5.6 in Australia, 5.5 in Canada, 5.2 in Finland [1]. The cervical cancer mortality rate (per 100,000 population) in Serbia was 7.9 in 2020, higher than the rates in developed countries: 2.1 in the United States, 1.9 in Canada, 1.5 in Australia, 1.1 in Finland [1]. The Serbian government recommends that women aged 20–65 years regularly undergo cervical cancer screening test [3], the purpose being early detection and treatment. Still, some women who receive a positive Papanicolaou (Pap) screening result do not undergo the recommended follow-up procedures [4,5]. Positive (that is, abnormal) Pap test result includes atypical squamous cells of undetermined significance (ASC-US), atypical glandular cells (AGC), low-grade squamous intraepithelial lesions (LSIL), high-grade squamous intraepithelial lesions (HSIL), adenocarcinoma in situ (AIS), and cervical cancer cells (squamous cell carcinoma or adenocarcinoma) [6].

Anxiety is often responsible for failing to adhere to follow-up procedures [7,8]. Achieving the largest coverage with quality screening and treatment is crucial for a program to be effective. It is challenging for developing but also for developed countries to achieve high cervical cancer screening coverage rates [9]. In the United States, 13% of invasive cervical cancer cases could have been avoided if women with a positive Pap smear had responded to prescribed diagnostic procedures [10]. However, the knowledge of adverse psychological effects of colposcopy and associated diagnostics is scarce [11,12].

Identifying women at risk for anxiety can significantly increase coverage by a medical procedure and significantly improve probability of survival. Investigation of predictors of anxiety in women with a positive Pap screening test prior to and following diagnostics is key for improving disease outcomes. A search of the available literature yielded no results regarding the use of ANN models for identifying women at risk for anxiety prior to and following diagnostics once receiving a positive Pap screening test. This study used ANN models because of their ability to model complex (both linear and non-linear) relationships between variables and provide more accurate predictions. The aim of the present study was to identify anxiety predictors prior to and following diagnostics in women who received a positive Pap screening result, and to build predictive models using ANNs. The hypothesis of this study is that ANNs built, trained and validated on surveyed data have the capability to precisely and accurately predict anxiety before and after diagnostic follow-up procedures in women with a positive Pap screening test.

## Materials and methods

### Setting

The research was done at the Clinical Center Kragujevac, a large University hospital in Serbia. Women who received a positive Pap smear within the screening program were directed, within four to six weeks, to additional diagnostic procedures at the Clinic of Gynecology and Obstetrics at the Clinical Center Kragujevac (where consultative colposcopy/biopsy/endocervical curettage are performed).

### Study design

The research was designed as a cross-sectional study, conducted prior to and following diagnostics in a cohort of women who have a positive Pap screening result.

### Study sample

Sample included all consecutive woman who received a positive Pap screening test and underwent diagnostic examination. Eligible women were 20–65 years old, with a Pap smear taken routinely within the population screening program that was abnormal in one previous year, with residency in Kragujevac district and fluency in spoken and written Serbian. Women were not eligible if aged < 20 and > 65, if pregnant in the moment of recruitment, if they previously underwent treatment for cervical lesions or had neuro-psychiatric disorders hindering study participation. Exclusion criteria were presence of diseases of reproductive organs with ongoing treatment in the moment of study, and refusal to participate.

### Sample size calculation

In women with a positive Pap screening test, Sharp et al. [12] found prevalence of anxiety according to the HADS (score ≥8 on the HADS anxiety subscale), to be 7.9% prior to and 23.7% following diagnostics. Using the software Epi Info Version 7.2.0.1 (Centers for Disease Control and Prevention, Atlanta, Georgia) with the Fleiss’s formula with continuity correction, α = 0.05 and desired study strength 95%, the minimum size of the sample was estimated to be 166.

Out of 238 eligible women, sample included 172 (response rate: 72.3%). Despite all efforts, 66 participants could not be included in the re-survey after 2–4 weeks, because they did not personally show up to receive their pathohistological examination results at the scheduled time.

### Data collection

A self-reported questionnaire was given to all participants prior to diagnostic examinations, along with a letter with information and written consent form. The first survey was performed immediately before the diagnostic examinations (consultative colposcopy / biopsy / endocervical curettage) were performed. The second survey was conducted 2–4 weeks after the mentioned diagnostics, i.e. immediately before obtaining the results of the diagnostic examinations. Surveys were conducted in the outpatient clinic of the Gynecology and Obstetrics Clinic of the Clinical Center Kragujevac. Participants had approximately 20(± 5) minutes to complete the survey.

### Instruments

The socio-demographic questionnaire collected data on participants’ age (≤30 / 31–40 / 41–50 / 51–60 / ≥61), place of residence (Rural / Urban), education level (≤ 8 years / > 8 years) and marital status (Unmarried / Married / Divorced, Widowed). Data on menopausal status, smoking habit, alcohol consumption, use of sedatives, family history for cervical cancer, personal history of other cancers, and history of anxiety and depression were also collected. This research also used the following instruments: "Hospital Anxiety and Depression Scale" (HADS) [13], “Cervical Dysplasia Distress Questionnaire” (CDDQ) [14], "The Center for Epidemiologic Studies Depression scale" (CES-D) [15], and "Process and Outcome Specific Measure" (POSM) [16].

The HADS is a self-reported screening tool that is used to identify and quantify anxiety and depression [13]. The HADS involves 14 items that represent two subscales: items relating to anxiety, HADS-A (7 items) and items relating to depression, HADS-D (7 items) over the last week. Agreement with each item is scored on a 4-point scale (0–3), to best describe how often participant felt in a particular way. Separate scoring is performed for anxiety and depression, with lower scores (0–7) denoting a normal level of anxiety/depression, and scores of 8–21 denoting presence of anxiety/depressive symptoms.

The HADS-A is used in assessing the presence and severity of symptoms of anxiety in clinical and general population. The purpose of the HADS-A is to screen for significant generalized anxiety symptoms. However, this result should not be interpreted as diagnostically significant for an anxiety disorder, even generalized anxiety disorder, but should be used to measure the presence of symptoms and to calibrate the severity of general anxiety [17]. Thus, the HADS-A provides broad coverage of general anxiety symptoms but does not provide features of specific anxiety disorders. The HADS-A includes specific items that assess generalized anxiety including tension, worry, fear, panic, difficulty relaxing, and restlessness [13,17]. In this study, internal consistency was high for the Serbian version of the HADS-A, with Cronbach’s alpha being 0.862 [18].

The CDDQ questionnaire [14] has 23 questions and 4 domains: the domains "Tension and discomfort" (6 items) and "Embarrassment" (2 items) measure psychological distress that is related to medical procedures (colposcopy), and domains "Concern about sexual and reproductive issues" (9 items) and "Concern about health consequences" (6 items) that measure distress that is related to consequences of getting a positive Pap smear test. The higher score indicates a higher distress.

The CES-D questionnaire screens for persons who are at risk of depression [15]. CES-D is a self-assessment scale of depressive symptoms, which are grouped into 4 domains: "Somatic complaints" (with 7 questions), "Positive affect" (with 4 questions), "Negative affect" (with 7 questions) and "Interpersonal relations" (with 2 questions). The scale’s score can be between 0 and 60, and if ≥16 it is considered as indicative of depression.

The POSM questionnaire [16] contains 14 questions related to awareness of one’s own abnormal Pap smear result, health concerns, satisfaction with support. The questions refer to the period between receiving the abnormal result and completing the questionnaire. A higher score of the POSM questionnaire indicated greater psychosocial burden.

None of the applied questionnaires were part of routine examination, and in no way influenced the course and outcome of diagnostic procedures. Current license holders provided consent for questionnaires’ use, and questionnaires were adapted linguistically and validated [18–20].

### Statistical analysis

Descriptive statistics was used to report variables as absolute numbers and frequencies, with the χ^2^-test used for comparison. Analyses were run in the SPSS software (The Statistical Package for Social Sciences software, SPSS Inc, version 20.0, Chicago, IL). Significance level of 95% (p<0.05) was defined for all tests.

In order to create an artificial neural network prediction model, the ANN that was used was a feedforward back-propagation multilayer perceptron, a supervised learning algorithm. The multilayer perceptron enabled modeling of nonlinear relationships between the characteristics of women who received a positive Pap screening test and occurrence of anxiety before and after the follow-up diagnostic procedures to which women were directed. Input variables were all of the collected respondents’ characteristics (socio-demographic and epidemiological). Dimensionality reduction was done to lower the probability of overfitting and increase generalizability of the created model, by reducing the number of features. For the selection of attributes that will be used to build the model, the "attribute selection" function was used to choose variables. The applied filter method is based on the correlation between the attributes and predicted class, i.e. between the input and output variables, and uses the Pearson’s coefficient with the Ranker search method. The output variables (classes), were presence or absence of anxiety at study time-point 1 for the ANN model that predicted pre-diagnostic anxiety, and presence or absence of anxiety at study time-point 2 for the ANN model that predicted post-diagnostic anxiety. The dataset splitting was performed, with 60% of data designated for training, 20% for validation and 20% for testing (external validation). Stratified cross-validation was done with 80% of data, while a set of 20% of the data was set aside as a test set. Hyperparameters of the ANN model (number of hidden layers, number of neurons in hidden layers, learning rate, momentum and number of epochs) were fine-tuned and adjusted using 10-fold cross-validation. The best performing model was constructed using the "trial & error" approach with the input of hyperparameters. Prediction performance of the created ANNs was assessed with the confusion matrix and the kappa statistic which indicated how correctly the outcome was predicted. Percentage of accuracy, rate of correctly classified positive outcomes, rate of false positive classified outcomes, precision, ROC (Receiver Operating Characteristic) area and Matthews correlation coefficient served for model performance evaluation. Data exploration and model building were performed in Waikato Environment for Knowledge Analysis (Weka, version 3.8.0, Waikato, New Zealand) software.

### Ethical considerations

This study is a part of research that was approved by the Ethics Committee of the Faculty of Medical Sciences, University of Kragujevac (Ref. No.: 01–2176) and by the Ethics Committee of the Clinical Center Kragujevac (Ref. No.: 01–2869). All participants provided written informed voluntary consent prior to taking part in the study and confidentiality was protected. The study recruitment period was between January 1^st^ 2017 and December 31^st^ 2017.

## Results

Nearly a third of women were between 51 and 60 years old (Table 1). Over 70% of respondents had urban place of residence. Almost 80% of women completed high school or higher education. Over 80% of women were with partner at the time of the study. Almost 40% of respondents were in menopause. More than half (57.0%) of the respondents were smokers, while 17.4% reported alcohol use. The frequency of symptoms indicating existence of anxiety was higher after diagnostic procedures (60.1%) than before (50.0%); difference in the frequency of anxiety following diagnostics was statistically significant (p = 0.030). Before the diagnostic procedures, women with a positive result of the Pap screening test who had anxiety were mostly in the fifth and sixth decade of life (p = 0.042). After the diagnostic procedures, women with a positive result of the Pap screening test who had anxiety were mostly with rural residence (p = 0.001). Women who reported anxiety in personal medical history were more likely to have anxiety after diagnostic procedures (p = 0.037), while those who reported depression in personal medical history were more likely to have anxiety before diagnostic procedures (p = 0.015). Women who reported the use of oral contraceptives were more likely to have anxiety both before and after diagnostic procedures (<0.001 and p = 0.041, respectively). The frequency of symptoms of depression (according to the CES-D scale, with a score ≥16) was significantly higher prior to diagnostic procedures (60.5%) than after diagnostics (48.1%), p<0.001). The difference in prevalence of depression (according to the HADS scale, with a score 8–21) prior to (68.8%) and following the diagnostics (75.7%) showed statistical significance (p<0.001).

**Table 1:** Women (N = 172) with abnormal Papanicolaou smear results: Socio-demograpfic characteristics and prevalence of anxiety (according to HADS-A subscale) before and after diagnostic procedures.

	Anxiety–present			
Variables	Before diagnostic procedures		After diagnostic procedures	
	Number (%)	P*	Number (%)	P*
**Age** (years)				
• ≤30	5 (5.8)		6 (5.7)	
• 31–40	15 (17.4)		24 (22.6)	
• 41–50	25 (29.1)		31 (29.2)	
• 51–60	26 (30.2)		33 (31.1)	
• ≥61	15 (17.4)	0.042	12 (11.3)	0.798
**Place of residence**				
• Rural	25 (29.1)		38 (35.8)	
• Urban	61 (70.9)	0.492	68 (64.2)	0.001
**Education level**				
• ≤8 years	21 (24.4)		23 (21.7)	
• >8 years	65 (75.6)	0.355	83 (78.3)	0.940
**Marital status**				
• Without partner	18 (20.9)		18 (17.0)	
• With partner	68 (79.1)	0.562	88 (83.0)	0.353
**Menopause**				
• No	48 (55.8)		60 (56.6)	
• Yes	38 (44.2)	0.214	46 (43.4)	0.191
**Cigarettes use**				
• No	37 (43.0)		43 (40.6)	
• Yes	49 (57.0)	1.000	63 (59.4)	0.411
**Alcohol consumption**				
• No	70 (81.4)		91 (85.8)	
• Yes	16 (18.6)	0.689	15 (14.2)	0.151
**Personal history of other cancer**				
• No	80 (93.0)		98 (92.5)	
• Yes	6 (7.0)	0.755	8 (7.5)	0.435
**Personal history for anxiety**				
• No	84 (97.7)		93 (87.7)	
• Yes	2 (2.3)	0.156	13 (12.3)	0.037
**Personal history for depression**				
• No	74 (86.0)		105 (99.1)	
• Yes	12 (14.0)	0.015	1 (0.9)	0.734
**Use of sedatives**				
• No	34 (53.1)		66 (62.3)	
• Yes	30 (46.9)	0.001	40 (37.7)	0.041
**Family history for cervical cancer**				
• No	76 (88.4)		94 (88.7)	
• Yes	10 (11.6)	0.445	12 (11.3)	0.425
**CES-D** (≥16 points)				
• No	34 (39.5)		55 (51.9)	
• Yes	52 (60.5)	<0.001	51 (48.1)	<0.001
**HADS-D** (8–21 points)				
• No	27 (31.4)		26 (24.5)	
• Yes	59 (68.8)	<0.001	80 (75.5)	<0.001
**Anxiety (HADS-A scor ≥8)**				
• No	86 (50.0)		66 (38.4)	
• Yes	86 (50.0)		106 (61.6)	0.030

Abbreviation: HADS-A (Hospital Anxiety and Depression Scale, Anxiety subscale), HADS-D (Hospital Anxiety and Depression Scale, Depression subscale); CES-D (the Center for Epidemiologic Studies Depression). P (*χ^2^-test).

**Fig 1:**
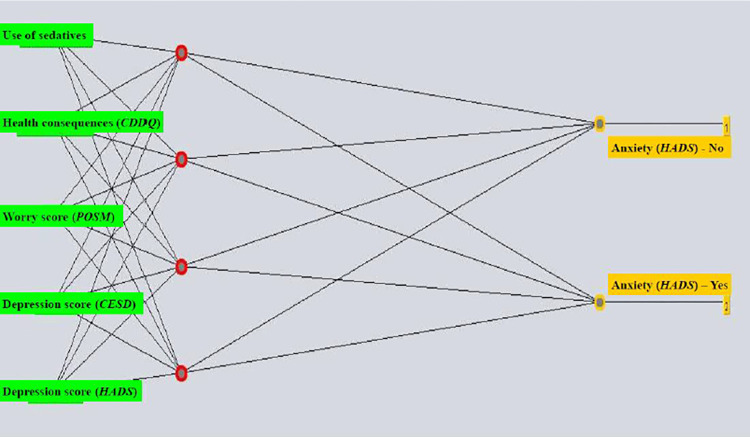
The multilayer perceptron structure: An artificial neural network model with most significant attributes selected for predicting anxiety (HADS-scale) in women with positive Pap results prior to diagnostic procedures. Abbreviations: HADS (Hospital Anxiety and Depression Scale); CDDQ (Cervical Dysplasia Distress Questionnaire); CES-D (the Center for Epidemiologic Studies Depression); POSM (Process and Outcome Specific Measure).

The pre-diagnostics anxiety ANN model parameters involved a learning rate of 0.6 with a momentum of 0.1 and 500 epochs and half the sum of the number of attributes and the number of classes as the number of neurons in the hidden layer (the model with all attributes). To construct the model with the selected attributes, a learning rate of 0.6 with a momentum of 0.1 and 500 epochs and 7 neurons in the hidden layer was applied. The Fig 1 shows ANN structure.

Attribute selection singled out 5 variables most significant for predicting anxiety among women with a positive Pap screening result prior to diagnostics: HADS score for depression, CESD score, subscale score Worry by the POSM scale, score for Concern about health consequences subscale score, and use of sedatives. Table 2 shows parameters for evaluating the model for predicting anxiety prior to diagnostics. Selecting attributes associated with the predicted outcome yields models that are more accurate in prediction compared to those models that employ all attributes. The model for predicting anxiety prior to diagnostics had a sensitivity of 76.5%, with a specificity of 75.0%. According to the area under the ROC curve (AUC), the model for predicting anxiety prior to diagnostics is a very good classifier (AUC 0.868).

**Table 2:** Metrics for evaluating the classification model for predicting anxiety (according to the HADS-scale) among women with positive Papanicolaou results prior to diagnostics.

Evaluation metrics	Model: Training +validation set** with all attributes	Model: Test set with all attributes	Model: Training + validation set** with selected attributes	Model: Test set with selected attributes
**Accuracy**	69.565%	67.647%	81.884%	76.471%
**Kappa**	0.389	0.310	0.638	0.514
**TP Rate** *	0.696	0.676	0.819	0.765
**FP Rate** *	0.307	0.376	0.180	0.250
Precision* **(PPV)**	0.695	0.671	0.820	0.765
**NPV**	0.703	0.636	0.841	0.714
**ROC Area** *	0.714	0.679	0.855	0.868
**MCC**	0.390	0.316	0.638	0.514

Abbreviations: HADS (Hospital Anxiety and Depression Scale); TP (True Positive rate); FP (False Positive rate); PPV (Positive Predictive Value); NPV (Negative Predictive Value); MCC (Matthews`correlation coefficient)

* pondered arithmetic mean for both classes

** 10-fold cross validation.

**Fig 2:**
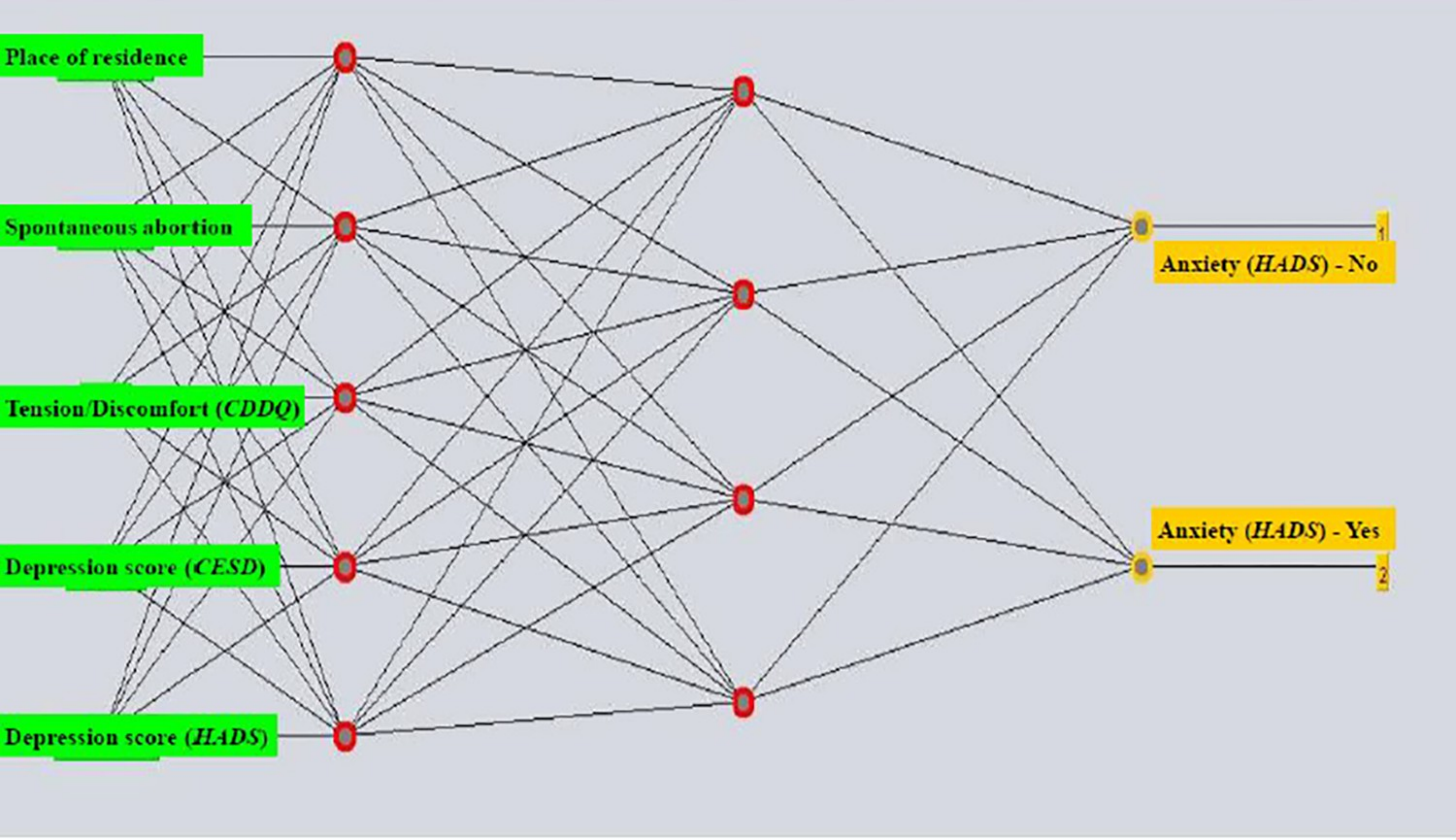
The multilayer perceptron structure: An artificial neural network model with most significant attributes selected for predicting anxiety (HADS-scale) in women with positive Pap results following diagnostic procedures. Abbreviations: HADS (Hospital Anxiety and Depression Scale); CDDQ (Cervical Dysplasia Distress Questionnaire); CES-D (the Center for Epidemiologic Studies Depression).

The post-diagnostic anxiety prediction ANN model involved: a learning rate of 0.6, momentum of 0.1, 1000 epochs and the sum of the number of attributes and the number of classes as the number of neurons in the hidden layer (the model with all attributes). To construct a model with selected attributes, a learning rate of 0.4, momentum of 0.3, 1000 epochs and 5 and 4 neurons in two hidden layers, respectively, were used. The Fig 2 shows the ANN structure. There were 5 variables singled out as the most significant attributes for predicting post-diagnostics anxiety among women with a positive Pap screening result: HADS-D score, CESD score, place of residence, spontaneous abortion and score on the Tension domain within the CDDQ. The model for predicting post-diagnostics anxiety showed sensitivity of 85.3% and a specificity of 79.0% (Table 3). The probability that a woman for whom the model predicts that she will develop anxiety actually develops anxiety is 88.2%. The excellent characteristics of the model as a classifier are reflected in the ROC value of 1.000, the high value of the Kappa statistic (0.679) and the Matthews correlation coefficient.

**Table 3:** Metrics for evaluating the classification model for predicting anxiety (according to the HADS-scale) among women with positive Papanicolaou results following diagnostics.

Evaluation metrics	Model: Training + validation set** with all attributes	Model: Test set with all attributes	Model: Training + validaton set** with selected attributes	Model: Test set with selected attributes
**Accuracy**	65.942%	64.706%	85.507%	85.294%
**Kappa**	0.261	0.184	0.696	0.679
**TP Rate** *	0.659	0.647	0.855	0.853
**FP Rate** *	0.404	0.483	0.148	0.210
Precision* **(PPV)**	0.654	0.681	0.858	0.882
**NPV**	0.714	0.633	0.902	0.800
**ROC Area** *	0.752	0.754	0.887	1.000
**MCC**	0.262	0.251	0.697	0.717

Abbreviations: HADS (Hospital Anxiety and Depression Scale); TP (True Positive rate); FP (False Positive rate); PPV (Positive Predictive Value); NPV (Negative Predictive Value); MCC (Matthews`correlation coefficient).

* pondered arithmetic mean for both classes

** 10-fold cross validation.

## Discussion

Findings of our study showed that predictors of anxiety among women who were attending follow-up diagnostics (consultative colposcopy/biopsy/endocervical curettage) after receiving a positive Pap screening test were depression, worry, concern about health consequences, use of sedatives, rural place of residence, spontaneous abortion in personal medical history, and tension/discomfort during colposcopy.

Unfortunately, to the best of our knowledge the available worldwide literature does not have reports of similar research. The ANNs have not been used for predicting anxiety prior to and following diagnostics that women with a positive Pap screening result are supposed to undergo. The ANN models have been used for predicting postmanic depression [21], depression in a geriatric population in India [22], postpartum depression in a population of pregnant women in Spain [23]. Research conducted in America [24] showed that the ANNs applied for predicting different psychological disorders after concussion, such as anxiety and depression, had an average accuracy of around 82.35%, consistent with accuracy obtained in our research. The application of ANNs in the field of cervical cancer research has mostly revolved classification of cytological findings [25,26] and survival estimation [27]. Namely, ANNs in medicine have most extensively been used in cervical cytopathology: the PAPNET system (PAPNET system, Cytologic Screening System for Quality Control of Cervical Smears; Neuromedical Systems, Inc., Suffern, NY) is an automated system for cytological screening of cervical smears, which shows better or equal accuracy compared to viewing under a light microscope [28].

A significant, powerful predictor of both pre- and post-diagnostics anxiety among women who received a positive Pap screening test was high score of depressive symptoms. Similar to this study, there were studies in women who received abnormal cervical cytology results that pointed out the significant association between anxiety and depression and undergoing follow-up diagnostics [29,30]. The association prior to diagnostics can be due to uncertainty, fear and expecting unknown negative outcomes, while post-diagnostics it can be related to possible adverse effects of colposcopy/biopsy/endocervical curettage (i.e. pain, bleeding, discharge) or other circumstances that women might be exposed to while waiting for the results (i.e. information via media, the environment, lack of support, etc.). A higher level of anxiety after diagnostics indicated the possible influence of some other variables which were not investigated in our study, and which were pointed out by some authors (such as the intention to have offspring, concern about HPV infection, etc.) [31].

The TOMBOLA study revealed that in women who have a positive pap screening test a higher worry level based on POSM scale significantly predicted adverse psychological outcomes prior to and following diagnostics [32], a finding that our research also confirmed. According to qualitative research, high anxiety prior to diagnostics that are follow-up to a positive Pap screening test is related to fear of cancer, fear of pain during the examination, and not being satisfied with information they received [31,33]. Apart from that, some research showed an association between higher anxiety in women prior to diagnostics and worry about the procedure itself, uncertainty of what examination involves and worry about pain, and worry about the outcome [34].

To the best of our knowledge, use of sedatives has not been previously singled out as a significant predictor of pre-diagnostics anxiety in women who have a positive Pap screening smear. The research so far has mostly explored anxiety disorders and use of sedatives in screening for cervical cancer and in receipt of abnormal results [35], and also during disease treatment [36]. Recently, findings of a meta-analysis that explored global disparities in cancer screening between people who have mental health issues and the general population found that women who had any mental health illness attended screening for cervical cancer significantly less than the general population [37]. The association between the use of sedatives and anxiety that we found could be explained by recent implementation of an organized cervical screening program in Serbia, insufficient information among women about what do the results of screening tests mean, as well as about the further examinations.

Disparities in colposcopy adherence between rural and urban areas exist and result in more advanced stages at presentation, and higher cervical cancer mortality rates in women living in rural areas [38,39]. Residing in a city could predict lower levels of post-diagnotic anxiety via an association with some other, potential predictors, which have not been confirmed as significant in our study (i.e. receiving information about the result of the Pap test, knowledge of the meaning of dysplasia, level of education and occupation). At the same time, urban way of life is mirrored in easier access to health services, easier transportation, more access to information, all that might serve as the reason for a negative association between living in the city and post-diagnostics anxiety level [40,41]. Besides, residing in a rural place as a predictor of higher post-diagnostic anxiety could be due to an association with some other, potential predictors not collected in our study (i.e. awareness of Pap test results, understanding cervical screening, knowledge about risk factors for cervical cancer, etc).

In this research, a higher level of post-diagnostic anxiety was noted in women who had anamnestic information about a spontaneous abortion. The literature shows that after an early pregnancy loss, women experience high levels of stress, anxiety, and depression [42,43]. It could be that spontaneous abortion serves as a predictor of post-diagnostic anxiety due to a possible link to disturbances in the hormonal milieu, infertility, and even psychological symptoms of depression and anxiety [42–44]. Although only sparse scientific literature is available on the consequences associated with fetal loss, such as anxiety, some studies have noted that anxiety occurs in 20% of women after spontaneous abortion [45,46].

Among women who participated in this study, there was an association between tension and discomfort that were experienced during medical procedures and post-diagnostic anxiety. With regard to post-diagnostic anxiety, the literature shows that after colposcopy and biopsy the most often stated reasons by women involved a negative experience during the procedure itself, and worry about being able to have children, worry about having cancer or having sexual activities [29,30]. After undergoing colposcopy-biopsy, what concerned women the most were fear of cancer, fear of losing reproductive abilities and sexual activities, fear of medical procedures (colposcopy, biopsy) and fear that something is wrong with how their body functions [47].

For the Pap smear to serve as an effective screening test, especially for preventing invasive cervical cancer, the women have to respond adequately to the needed further diagnostic procedures and treatment [4,5,48]. Our research made it possible to reveal factors that are associated with pre- and post-diagnostic anxiety among women who received a positive Pap screening test result, and to create models that enable prediction of these women’s adverse psychological reactions, that might in turn have a positive impact on their participation in diagnostic procedures.

### Strengths and limitations of the research

Based on the available literature, until now there have been no studies that employed ANNs to create predictive models for pre- and pos-diagnostic anxiety in women who received a positive Pap screening test. The research presented in this study was the first to apply ANNs in developing models for predicting anxiety in a sample of Serbian women who received positive Pap screening results. The test subjects were included by recruitment at the only gynecological clinic for the entire population that is included in the cervical cancer screening, indicating that all participants had the same chance of participating. Consequently, the study sample was not selected, which suggests its representativeness for the entire study population which is included in cervical cancer screening. In this research, only questionnaires that are validated questionnaires were used [18–20].

Nevertheless, the present study has several limitations. Apart from the known shortcomings of the cross-sectional study design, using a self-report questionnaire is also a limitation. Of course, the sample size can always be questioned. The presence of information bias cannot be ruled out with certainty, since, even though the researchers guaranteed the privacy of all information, it can always remain possible that some respondents did not wish to disclose anxiety. Still, the anxiety of our subjects was estimated with a validated questionnaire, thus minimizing possible biases. Also, the question of whether the sample is representative can always be raised, due to barriers in engaging female participants in research or due to the fear of breach of confidentiality. Further, no clinical confirmation of anxiety and depression was present, nor was there insight into their medical records, except for findings related to cervical cancer screening. Besides, the limitation of this study is the lack of data on the psychological status of women before the Pap test, as well as after obtaining the abnormal Pap test result and referral to further diagnostic procedures. Also, this research did not provide information on other factors that could predict anxiety (i.e. socioeconomic status, concerns about HPV infection, status of sexual partners in terms of sexually transmitted diseases, etc.). Finally, the effect of some other factors that the subjects might have been exposed to in the meantime and that might affect the anxiety level pre- and post-diagnostics cannot be ruled out with certainty.

### Implications

To address the barriers to adherence after an abnormal Papanicolaou smear in the anxious women is of considerable public health importance. Several studies investigating how to reduce anxiety and increase adherence to diagnostic procedures in women who received a positive screening test for cervical cancer have produced contradictory results. Previous studies suggested that music during colposcopy significantly reduced anxiety levels and pain experienced during the procedure [49]. In contrast, recent systematic reviews and meta-analyses have shown that music therapy, educational intervention through leaflets, mail, phone, or video information had no positive effect in reducing anxiety levels during colposcopy in women with abnormal cervical cytology [50–52]. One qualitative study indicated that women with abnormal Pap smear results required individualized oral information [53]. Also, one trial in Thailand showed that colposcopy counseling was effective in reducing anxiety in women with abnormal cervical cytology results [54]. Therefore, colposcopy counseling and positive interactions between patient and health professionals are helpful the most in relieving anxiety in women during their colposcopy attendance.

## Conclusions

The presented findings can aid healthcare providers in deciding for the necessity of psychological support for women who have received a positive Pap screening test, thus enabling inclusion of as many women as possible in the follow-up diagnostics, timely initiation of treatment and reduction in morbidity and mortality from cervical cancer.

## References

[1] Singh D, Vignat J, Lorenzoni V, Eslahi M, Ginsburg O, Lauby-Secretan B, et al. Global estimates of incidence and mortality of cervical cancer in 2020: a baseline analysis of the WHO Global Cervical Cancer Elimination Initiative. Lancet Glob Health. 2023; 11(2): e197–e206 doi: 10.1016/S2214-109X(22)00501-0 36528031 PMC9848409

[2] Sung H, Ferlay J, Siegel RL, Laversanne M, Soerjomataram I, Jemal A, et al. Global Cancer Statistics 2020: GLOBOCAN Estimates of Incidence and Mortality Worldwide for 36 Cancers in 185 Countries. CA Cancer J Clin. 2021; 71(3): 209–249 doi: 10.3322/caac.21660 33538338

[3] Ilic M, Ilic I. Cancer mortality in Serbia, 1991–2015: an age-period-cohort and joinpoint regression analysis. Cancer Commun (Lond). 2018; 38(1): 10 doi: 10.1186/s40880-018-0282-3 29764495 PMC5993142

[4] Miller SM, Tagai EK, Wen KY, Lee M, Hui SA, Kurtz D, et al. Predictors of adherence to follow-up recommendations after an abnormal Pap smear among underserved inner-city women. Patient Educ Couns. 2017; 100(7): 1353–1359 doi: 10.1016/j.pec.2017.01.020 28190541 PMC5466500

[5] Petersen Z, Jaca A, Ginindza TG, Maseko G, Takatshana S, Ndlovu P, et al. Barriers to uptake of cervical cancer screening services in low-and-middle-income countries: a systematic review. BMC Womens Health. 2022; 22(1): 486 doi: 10.1186/s12905-022-02043-y 36461001 PMC9716693

[6] Solomon D, Davey D, Kurman R, Moriarty A, O’Connor D, Prey M, et al; Forum Group Members; Bethesda 2001 Workshop. The 2001 Bethesda System: terminology for reporting results of cervical cytology. JAMA. 2002; 287(16): 2114–2119 doi: 10.1001/jama.287.16.2114 11966386

[7] Sharp L, Cotton S, Cruickshank M, Gray N, Smart L, Whynes D, et al; TOMBOLA Group. Impact of post-colposcopy management on women’s long-term worries: results from the UK population-based TOMBOLA trial. J Fam Plann Reprod Health Care. 2016; 42(1): 43–51 doi: 10.1136/jfprhc-2015-101170 26376822

[8] Martinez-Gutierrez J, Chima S, Boyd L, Sherwani A, Drosdowsky A, Karnchanachari N, et al. Failure to follow up abnormal test results associated with cervical cancer in primary and ambulatory care: a systematic review. BMC Cancer. 2023; 23(1): 653 doi: 10.1186/s12885-023-11082-z 37438686 PMC10337158

[9] Gakidou E, Nordhagen S, Obermeyer Z. Coverage of cervical cancer screening in 57 countries: low average levels and large inequalities. PLoS Med. 2008; 5(6): e132 doi: 10.1371/journal.pmed.0050132 18563963 PMC2429949

[10] Leyden WA, Manos MM, Geiger AM, Weinmann S, Mouchawar J, Bischoff K, et al. Cervical cancer in women with comprehensive health care access: attributable factors in the screening process. J Natl Cancer Inst. 2005; 97(9): 675–683 doi: 10.1093/jnci/dji115 15870438

[11] Adegboyega A, Dignan M, Sha S, Nkwonta C, Williams LB. Psychological factors among Appalachian women with abnormal Pap results. J Rural Health. 2022; 38(2): 382–390 doi: 10.1111/jrh.12585 33955052 PMC8571115

[12] Sharp L, Cotton S, Little J, Gray NM, Cruickshank M, Smart L, et al; TOMBOLA Group. Psychosocial impact of alternative management policies for low-grade cervical abnormalities: results from the TOMBOLA randomised controlled trial. PLoS One. 2013; 8(12): e80092 doi: 10.1371/journal.pone.0080092 24386076 PMC3875419

[13] Zigmond AS, Snaith RP. The hospital anxiety and depression scale. Acta Psychiatr Scand. 1983; 67(6): 361–370 doi: 10.1111/j.1600-0447.1983.tb09716.x 6880820

[14] Shinn E, Basen-Engquist K, Le T, Hansis-Diarte A, Bostic D, Martinez-Cross J, et al. Distress after an abnormal Pap smear result: scale development and psychometric validation. Prev Med. 2004; 39(2): 404–412 doi: 10.1016/j.ypmed.2004.02.004 15226053

[15] Radloff LS. The CES-D Scale: a self-report depression scale for research in the general population. Appl Psychol Measurement. 1977; 1: 385–401 doi: 10.1177/014662167700100306

[16] Gray NM, Sharp L, Cotton SC, Avis M, Philips Z, Russell I, et al; TOMBOLA group. Developing a questionnaire to measure the psychosocial impact of an abnormal cervical smear result and its subsequent management: the TOMBOLA (Trial of Management of Borderline and Other Low-grade Abnormal Smears) trial. Qual Life Res. 2005; 14(6): 1553–1562 doi: 10.1007/s11136-004-8146-5 16110935

[17] Wall AD, Lee EB. What do anxiety scales really measure? An item content analysis of self-report measures of anxiety. J Psychopathol Behav Assess. 2022; 44(4): 1148–1157 doi: 10.1007/s10862-022-09973-9

[18] Ilic I, Babic G, Dimitrijevic A, Ilic M, Sipetic Grujicic S. Internal consistency and validity of the Hospital Anxiety and Depression Scale (HADS) in women with abnormal Pap smear in Serbia. Women Health. 2021; 61(4): 363–371 doi: 10.1080/03630242.2021.1893244 33641629

[19] Ilic I, Babic G, Dimitrijevic A, Ilic M, Sipetic Grujicic S. Psychological distress among women with abnormal pap smear results in Serbia: Validity and reliability of the Cervical Dysplasia Distress Questionnaire. PLoS One. 2019; 14(6): e0218070 doi: 10.1371/journal.pone.0218070 31188876 PMC6561558

[20] Ilic I, Babic G, Dimitrijevic A, Ilic M, Sipetic Grujicic S. Reliability and validity of the Center for Epidemiologic Studies Depression (CES-D) scale in Serbian women with abnormal Papanicolaou smear results. Int J Gynecol Cancer. 2019;.29(6):.996–1002 doi: 10.1136/ijgc-2019-000219 31203200

[21] Jefferson MF, Pendleton N, Lucas CP, Lucas SB, Horan MA. Evolution of artificial neural network architecture: prediction of depression after mania. Methods Inf Med. 1998; 37(03): 220–225 doi: 10.1055/s-0038-1634532 9787620

[22] Sau A, Bhakta I. Artificial neural network (ANN) model to predict depression among geriatric population at a slum in Kolkata, India. J Clin Diagn Res. 2017; 11(5): VC01–VC04 doi: 10.7860/JCDR/2017/23656.9762 28658883 PMC5483785

[23] Tortajada S, García-Gomez JM, Vicente J, Sanjuán J, de Frutos R, Martín-Santos R, et al. Prediction of postpartum depression using multilayer perceptrons and pruning. Methods Inf Med. 2009; 48(3): 291–298 doi: 10.3414/ME0562 19387507

[24] Dabek F, Caban JJ. A neural network based model for predicting psychological conditions. In: Guo Y, Friston K, Aldo F, Hill S, Peng H, editors. Brain Informatics and Health. BIH 2015. Lecture Notes in Computer Science, Vol. 9250. Cham: Springer. doi: 10.1007/978-3-319-23344-4_25

[25] Bengtsson E, Malm P. Screening for cervical cancer using automated analysis of PAP-smears. Comput Math Methods Med. 2014; 2014: 842037 doi: 10.1155/2014/842037 24772188 PMC3977449

[26] Koss LG, Lin E, Schreiber K, Elgert P, Mango L. Evaluation of the PAPNET cytologic screening system for quality control of cervical smears. Am J Clin Pathol. 1994; 101(2): 220–229 doi: 10.1093/ajcp/101.2.220 8116579

[27] Ochi T, Murase K, Fujii T, Kawamura M, Ikezoe J. Survival prediction using artificial neural networks in patients with uterine cervical cancer treated by radiation therapy alone. Int J Clin Oncol. 2002; 7(5): 294–300 doi: 10.1007/s101470200043 12402063

[28] Pouliakis A, Karakitsou E, Margari N, Bountris P, Haritou M, Panayiotides J, et al. Artificial Neural Networks as Decision Support Tools in Cytopathology: Past, Present, and Future. Biomed Eng Comput Biol. 2016; 7: 1–18 doi: 10.4137/BECB.S31601 26917984 PMC4760671

[29] Hellsten C, Sjöström K, Lindqvist PG. A prospective Swedish cohort study on psychosocial factors influencing anxiety in women referred for colposcopy. BJOG. 2007; 114(1): 32–38 doi: 10.1111/j.1471-0528.2006.01161.x 17233857

[30] Gray NM, Sharp L, Cotton SC, Masson LF, Little J, Walker LG, et al; TOMBOLA group. Psychological effects of a low-grade abnormal cervical smear test result: anxiety and associated factors. Br J Cancer. 2006; 94(9): 1253–1262 doi: 10.1038/sj.bjc.6603086 16622462 PMC2361408

[31] O’Connor M, Waller J, Gallagher P, Martin CM, O’Leary JJ, D’Arcy T, et al; Irish Screening Research Consortium (CERVIVA). Understanding Women’s Differing Experiences of Distress after Colposcopy: A Qualitative Interview Study. Womens Health Issues. 2015; 25(5): 528–534 doi: 10.1016/j.whi.2015.05.009 26189936

[32] Cotton SC, Sharp L, Little J, Gray NM, Walker LG, Whynes DK, et al; TOMBOLA Group. A normal colposcopy examination fails to provide psychological reassurance for women who have had low-grade abnormal cervical cytology. Cytopathology. 2015; 26(3): 178–187 doi: 10.1111/cyt.12173 25099940

[33] Monsonego J, Cortes J, da Silva DP, Jorge AF, Klein P. Psychological impact, support and information needs for women with an abnormal Pap smear: comparative results of a questionnaire in three European countries. BMC Womens Health. 2011; 11: 18. doi: 10.1186/1472-6874-11-18 21612599 PMC3123641

[34] Bosgraaf RP, de Jager WC, Servaes P, Prins JB, Massuger LF, Bekkers RL. Qualitative insights into the psychological stress before and during colposcopy: a focus group study. J Psychosom Obstet Gynaecol. 2013; 34(4): 150–156 doi: 10.3109/0167482X.2013.849688 24188786

[35] Zhang X, Mkuu R, Samman E, Cummings S, Sherman L, Wigfall LT, et al. Anxiety and depressive symptoms and missing breast cancer and cervical screening: results from Brazos valley community health survey. Psychol Health Med. 2020; 25(4): 402–409 doi: 10.1080/13548506.2019.1668031 31532238

[36] Horsbøl TA, Kjaer SK, Andersen EW, Ammitzbøll G, Thygesen LC, Johansen C, et al. Use of hypnotics among women diagnosed with cervical cancer—A population-based cohort study. Gynecol Oncol. 2022; 166(2): 300–307 doi: 10.1016/j.ygyno.2022.05.019 35680430

[37] Solmi M, Firth J, Miola A, Fornaro M, Frison E, Fusar-Poli P, et al. Disparities in cancer screening in people with mental illness across the world versus the general population: prevalence and comparative meta-analysis including 4 717 839 people. Lancet Psychiatry. 2020; 7(1): 52–63 doi: 10.1016/S2215-0366(19)30414-6 31787585

[38] Percac-Lima S, Aldrich LS, Gamba GB, Bearse AM, Atlas SJ. Barriers to follow-up of an abnormal Pap smear in Latina women referred for colposcopy. J Gen Intern Med. 2010; 25(11): 1198–1204 doi: 10.1007/s11606-010-1450-6 20652647 PMC2947627

[39] Kohler RE, Hemler J, Wagner RB, Sullivan B, Macenat M, Tagai EK, et al. Confusion and anxiety in between abnormal cervical cancer screening results and colposcopy: "The land of the unknown". Patient Educ Couns. 2023; 114: 107810 doi: 10.1016/j.pec.2023.107810 37244133 PMC10527466

[40] Watkins MM, Gabali C, Winkleby M, Gaona E, Lebaron S. Barriers to cervical cancer screening in rural Mexico. Int J Gynecol Cancer. 2002; 12(5): 475–479 doi: 10.1046/j.1525-1438.2002.01170.x 12366665

[41] Suk R, Hong YR, Rajan SS, Xie Z, Zhu Y, Spencer JC. Assessment of US Preventive Services Task Force Guideline-Concordant Cervical Cancer Screening Rates and Reasons for Underscreening by Age, Race and Ethnicity, Sexual Orientation, Rurality, and Insurance, 2005 to 2019. JAMA Netw Open. 2022; 5(1): e2143582 doi: 10.1001/jamanetworkopen.2021.43582 35040970 PMC8767443

[42] Jung SJ, Shin A, Kang D. Hormone-related factors and post-menopausal onset depression: results from KNHANES (2010–2012). J Affect Disord. 2015; 175: 176–183 doi: 10.1016/j.jad.2014.12.061 25622021

[43] Hope H, Pierce M, Johnstone ED, Myers J, Abel KM. The sexual and reproductive health of women with mental illness: a primary care registry study. Arch Womens Ment Health. 2022; 25(3): 585–593 doi: 10.1007/s00737-022-01214-y 35366692 PMC9072520

[44] Tetruashvili N, Domar A, Bashiri A. Prevention of Pregnancy Loss: Combining Progestogen Treatment and Psychological Support. J Clin Med. 2023; 12(5): 1827 doi: 10.3390/jcm12051827 36902614 PMC10003391

[45] Neugebauer R. Depressive symptoms at two months after miscarriage: interpreting study findings from an epidemiological versus clinical perspective. Depress Anxiety. 2003; 17(3): 152–161 doi: 10.1002/da.10019 12768649

[46] Munk-Olsen T, Bech BH, Vestergaard M, Li J, Olsen J, Laursen TM. Psychiatric disorders following fetal death: a population-based cohort study. BMJ Open. 2014; 4(6): e005187 doi: 10.1136/bmjopen-2014-005187 24907247 PMC4054628

[47] Sharp L, Cotton S, Carsin AE, Gray N, Thornton A, Cruickshank M, et al; TOMBOLA Group. Factors associated with psychological distress following colposcopy among women with low-grade abnormal cervical cytology: a prospective study within the Trial Of Management of Borderline and Other Low-grade Abnormal smears (TOMBOLA). Psychooncology. 2013; 22(2): 368–380 doi: 10.1002/pon.2097 22162138

[48] Eggleston KS, Coker AL, Das IP, Cordray ST, Luchok KJ. Understanding barriers for adherence to follow-up care for abnormal pap tests. Journal Womens Health (Larchmt). 2007; 16(3): 311–330 doi: 10.1089/jwh.2006.0161 17439377

[49] Galaal K, Bryant A, Deane KH, Al-Khaduri M, Lopes AD. Interventions for reducing anxiety in women undergoing colposcopy. Cochrane Database Syst Rev. 2011;2011(12):CD006013 doi: 10.1002/14651858.CD006013.pub3 22161395 PMC4161490

[50] Mak N, Reinders IMA, Slockers SA, Westen EHMN, Maas JWM, Bongers MY. The effect of music in gynaecological office procedures on pain, anxiety and satisfaction: a randomized controlled trial. Gynecol Surg. 2017;14(1):14. doi: 10.1186/s10397-017-1016-2 28890676 PMC5570770

[51] Abdelhakim AM, Samy A, Abbas AM. Effect of music in reducing patient anxiety during colposcopy: A systematic review and meta-analysis of randomized controlled trials. J Gynecol Obstet Hum Reprod. 2019;48(10):855–861 doi: 10.1016/j.jogoh.2019.07.007 31276848

[52] Wouters T, Soomers J, Smink M, Smit RA, Plaisier M, Houterman S, et al. The effect of an animation video on consultation time, anxiety and satisfaction in women with abnormal cervical cytology: Animation video reduces colposcopy time. Prev Med Rep. 2019;13:238–243 doi: 10.1016/j.pmedr.2019.01.005 30719404 PMC6350223

[53] Rask M, Swahnberg K, Lindell G, Oscarsson M. Women’s experiences of abnormal Pap smear results—A qualitative study. Sex Reprod Healthc. 2017;12:3–8 doi: 10.1016/j.srhc.2017.01.002 28477928

[54] Pornsinsiriruck S, Sumdaengrit B, Kongrot S, Jengprasert K, Puntusopon N. The effect of colposcopy counseling with a feminist model on anxiety in Thai women with abnormal cervical cytology results: A time-series quasi-experimental study. Belitung Nurs J. 2023;9(6):611–618 doi: 10.33546/bnj.2924 38130668 PMC10731425

